# Supplementary material for: Sequencing of the invasive E. coli strain BEN2908 isolated from poultry: A comparative investigation of genomic regions shared with intestinal and extraintestinal model E. coli strains
Source: PLoS One. 2026 Feb 23;21(2):e0342894. doi: 10.1371/journal.pone.0342894 (PMC12928451; doi:10.1371/journal.pone.0342894)
Supplement: Attachment — Submitted filename: PONE-D-25-49371_reviewed.pdf [file pone.0342894.s008.pdf]

**Peer Review:** "Sequencing of the Invasive E. coli Strain BEN2908 Isolated from Poultry: A Comparative Investigation of Genomic Regions Shared with Other Invasive Model Strains" PONE-D-25-49371

**Recommendation: Major Revision**

**Reviewer Comments**

This is a very complete and carefully executed study. The authors present a full genome assembly and comparative analysis of the APEC strain BEN2908, with integration of plasmid features and comparison to other ExPEC and AIEC strains. The manuscript is generally well organized and within scope for PLOS ONE.

That said, to fully meet the journal's standards of technical rigor, reproducibility, and cautious interpretation, I recommend major revision.

**Global Comments**

The manuscript would benefit from a clearer description of the origin of strain BEN2908. Please specify the sample type, host species, geographic origin, year of isolation, and whether this strain was previously described or deposited in a public collection. These details are critical to contextualize the comparative analysis with other ExPEC and AIEC isolates.

The genomic methodology is described in commendable detail, but essential elements are still missing to evaluate assembly quality and reproducibility. In particular,

The Introduction is well written and easy to follow, but the narrative would be strengthened by briefly introducing the main virulence and metabolic genes that are later discussed in the Results (e.g., iron uptake systems, T6SS loci, dicarboxylate transporters). Providing this context early on would prepare the reader for the subsequent comparative analysis.

In the Methods, the description of the Illumina sequencing is too limited. While the platform (MiSeq), read number, length, and GC content are reported, key information is missing for reproducibility and assessment of data quality.

Please, provide coverage statistics across the chromosome and plasmid, QC metrics (e.g., FastQC), and details on any trimming or filtering steps prior to assembly. Given that the data were generated in 2015, it would also be important to clarify whether quality was re-evaluated before use in this study. Finally, to meet PLOS ONE's data availability requirements, the raw Illumina reads should be deposited in SRA with accession numbers included in the Data Availability Statement. Also, provide version/date for the web-based tools to ensure reproducibility (especially the tools that uses online databases).

The RAxML tree must include bootstrap support values. At present the conclusions about STc95 and STc135 are not supported.

Please specify the BLAST settings (identity, coverage thresholds, word size, or e-value) used to define the genomic regions (GRs), and justify the specific ≥4 kb cutoff, as this arbitrary threshold affects which loci are considered "GRs."

In addition, while Tables S1–S2 summarize GC content, coverage, and identities, the underlying files (BRIG project, FASTA sequences of each GR, and CDS functional annotation tables) should be deposited in a public repository (for example Github, to allow replication). If it is available, scripts should be public on GitHub or public repositories, or more detailed in a section of supplementary methods. In the Table 2 and Figure they some plasmids are analyzed but they are not available for download. If pLF82 data were taken from an older publication, the comparative analysis is only partially reproducible; please provide the accession numbers or upload the sequence to a public repository.

For the functional assignments, please clarify the criteria for selecting "reviewed" vs "unreviewed" UniProtKB hits and provide the accession numbers of representative proteins. Without these details, other researchers cannot reproduce or verify the classification of features.

Please temper the VFAnalyzer/PHASTEST claims (from lines 221) by specifying identity/coverage thresholds, depositing the full outputs (Tables S4–S5 with raw hits), and avoiding absolute statements like "shares all" without qualification. I The same situation is in the plasmid section (from line 237) the description of pBEN2908 is super clear, but the statement that "all genes related to a ColV-like plasmid are present" is too absolute without showing the evidence in detail. A table listing each ColV-associated locus (e.g., iuc/iut, iro, etc) with accession, coverage, and identity values would be useful and also the Inc or MOB type groups in the same table.

In the Sugar Metabolism section (from line 328), please distinguish more clearly between functions previously validated in the literature (e.g., frz operon, GimA) and novel pathway predictions based on low-identity ORFs, so that readers can separate established evidence from putative annotations and similar in the identification of iron and metal uptake systems (from line 404), the phrasing "we identified systems related to metal uptake" should be revised to clarify that this refers to the detection of previously described operons through genome annotation, rather than novel experimental findings. In the Adhesion and Invasion section (lines 412), please clarify that operons and loci (fim, auf, fdeC, ila) were detected through annotation rather than experimentally demonstrated, and temper the functional claims for ORF $1_{11}$ as putative predictions based on moderate homology.

The Discussion is very detailed and in parts can be excessive. Several subsections (e.g., Sugar Metabolism, General Metabolism) repeat pathway-level explanations that could be streamlined or moved to Supplementary Material. A more concise discussion, with selective emphasis on novel findings, would greatly improve readability. Consider moving some of the descriptive

detail into the Introduction (to set context) or into Supplementary Notes, while keeping the main text focused on the comparative insights.

In the Conclusion, please temper the statement on long-term conservation of uncharacterized ORFs, which is only inferred from two isolates 21 years apart, and explicitly clarify that all proposed functions remain hypothetical pending experimental validation.

**Minor revisons:**

The statement that these findings "reinforce the importance in government monitoring of bacterial genomic evolution" (line 276) introduces a policy-oriented opinion that goes beyond the scope of the presented data. I recommend rephrasing this sentence to remain focused on the scientific evidence (i.e., the role of ColV-like plasmids in virulence).

Replace "softwares" with "software," shorten long passive sentences.

Reframe causal or absolute statements ("shares all genes," "ancestral proximity") into cautious, data-supported wording ("shares X genes above threshold Y," "phylogenetic proximity suggested but requires bootstrap support").

**Specific Comments**

- **Line 71**: Please make explicit that you are now referring to human disease context.
- **Line 93**: Be careful with the term "cassette gene." Are you referring to integron cassettes or simply modular groups of mobilizable genes? Clarify the terminology.
- **Line 104**: Explain or cite the "minor modification" of the ONT nanopore protocol. Without details, it is not reproducible.
- **Line 120:** Please, provide the RAST version
- **Lines 113–115**: Wording issue: "The obtained raw data (.fast5 files) was base called…" → should be "The raw data were basecalled with Guppy v4.0.11 using [config]." Please also specify qscore filter and parameters.
- **Line 210–215**: The statement that STc95 is closer to AIEC STc135 than to APEC SCI-07 cannot stand without bootstrap support. Please provide node support and, ideally, ANI/AAI values.
- **Line 222–229**: When describing shared virulence genes with LF82, avoid absolute terms like "all 76 genes." Instead, specify thresholds for identity/coverage and include a full gene list in Supplementary Materials.
- **Line 300–311 (Table 3)**: Provide coverage/identity values for each "genomic region" absent or present, or the cut off used for declare as presence/absence.

- **Line 461–466**: The CRISPR comparison is interesting, but please provide a full spacer table with coordinates and system subtype classification to avoid confusion.
- **Line 650–656 (Conclusion)**: The wording "attests the utility and importance" is too strong. Suggest softening to "suggests possible functional utility, subject to experimental validation."

**Recommendation:** Major revision.

This is a very complex and beautifully crafted manuscript that will be a valuable reference once revised. With the additions of assembly QC, reproducibility deposits, phylogenetic support, careful terminology and parameters, the paper will fully meet the *PLOS ONE* publication criteria. Once these issues are addressed, the manuscript will meet the journal's standards for transparency, reproducibility, and cautious interpretation.
