# Supplementary material for: Sequencing of the invasive E. coli strain BEN2908 isolated from poultry: A comparative investigation of genomic regions shared with intestinal and extraintestinal model E. coli strains
Source: PLoS One. 2026 Feb 23;21(2):e0342894. doi: 10.1371/journal.pone.0342894 (PMC12928451; doi:10.1371/journal.pone.0342894)
Supplement: Attachment — Submitted filename: Review Report-PONE-D-49371.docx [file pone.0342894.s009.docx]

The manuscript “Sequencing of the Invasive E. coli Strain BEN2908 Isolated from Poultry: A

Comparative Investigation of Genomic Regions Shared with Other Invasive Model

Strains” by Martins et al., provide insight into sequence similarity between a invasive strain BEN2908 from poultry and compares it with previously reported E. coli genomes. The study identified sequence diversity as well as conserved regions in the genome and its biological significance. Following are the minor comments on the manuscript.

1. References for the genes that contribute to the virulence of E. coli (line 280) should be provided
2. In line 335, reference 21 appears in italics, and reference 153 is also italicized; please ensure consistent formatting.
3. The references numbered 154–159 are listed in the reference section but not cited in the main text.
4. At line 609, the temperature unit is incorrectly written as ‘Celsius’; it should be expressed in °C, consistent with the formatting used throughout the manuscript
5. **Please provide a reference for the previously described GRs in E. coli mentioned in line 413.**
6. **Please include an appropriate reference to support the statement in line 531.**
