# Supplementary material for: Sequencing of the invasive E. coli strain BEN2908 isolated from poultry: A comparative investigation of genomic regions shared with intestinal and extraintestinal model E. coli strains
Source: PLoS One. 2026 Feb 23;21(2):e0342894. doi: 10.1371/journal.pone.0342894 (PMC12928451; doi:10.1371/journal.pone.0342894)
Supplement: Attachment — Submitted filename: response reviewers PLOS ONE PONE-D-25-49371_final.docx [file pone.0342894.s011.docx]

# Response to Reviewers comments:

Reviewer #1: Peer Review: “Sequencing of the Invasive E. coli Strain BEN2908 Isolated from Poultry: A Comparative Investigation of Genomic Regions Shared with Other Invasive Model Strains” PONE-D-25-49371
Recommendation: Major Revision

Reviewer Comments

This is a very complete and carefully executed study. The authors present a full genome assembly and comparative analysis of the APEC strain BEN2908, with integration of plasmid features and comparison to other ExPEC and AIEC strains. The manuscript is generally well organized and within scope for PLOS ONE.
That said, to fully meet the journal’s standards of technical rigor, reproducibility, and cautious interpretation, I recommend major revision.

We thank the reviewer for these insightful and constructive comments, which have helped us to significantly improve our manuscript.

Global Comments

1. The manuscript would benefit from a clearer description of the origin of strain BEN2908. Please specify the sample type, host species, geographic origin, year of isolation, and whether this strain was previously described or deposited in a public collection. These details are critical to contextualize the comparative analysis with other ExPEC and AIEC isolates.

BEN2908 has been described in the introduction with the requested information (lines 70-73) and the following text was added in the Data availability section (lines 260-261) regarding deposition in a public collection: ”BEN2908 strain has been deposited at the International Center for Microbial Resources—Bacterial Pathogens (CIRM-BP) under name CIRMBP-1386.”

2. The genomic methodology is described in commendable detail, but essential elements are still missing to evaluate assembly quality and reproducibility. In particular, The Introduction is well written and easy to follow, but the narrative would be strengthened by briefly introducing the main virulence and metabolic genes that are later discussed in the Results (e.g., iron uptake systems, T6SS loci, dicarboxylate transporters). Providing this context early on would prepare the reader for the subsequent comparative analysis.

The Introduction was restructured to mention the importance of metal uptake in APEC infection, the recently shown relation of dicarboxylates in APEC virulence (lines 54-60), and the contribution of the T6SS to both APEC and AIEC pathogenicity (lines 102-106).

3. In the Methods, the description of the Illumina sequencing is too limited. While the platform (MiSeq), read number, length, and GC content are reported, key information is missing for reproducibility and assessment of data quality. Please, provide coverage statistics across the chromosome and plasmid, QC metrics (e.g., FastQC), and details on any trimming or filtering steps prior to assembly. Given that the data were generated in 2015, it would also be important to clarify whether quality was re-evaluated before use in this study. Finally, to meet PLOS ONE’s data availability requirements, the raw Illumina reads should be deposited in SRA with accession numbers included in the Data Availability Statement.

The requested information was added to the Methods “BEN2908 DNA extraction, sequencing, assembly and annotation” section (lines 143-151) and the sequencing files mentioned were submitted to GitHub, under the following repository: <https://github.com/Martins-TW/BEN2908_Genome_Analysis.git>

SRA files were submitted to BioProject PRJNA1359407 (lines 258-259).

4. Also, provide version/date for the web-based tools to ensure reproducibility (especially the tools that uses online databases).

All versions and releases were added to their respective Methods sections.

5. The RAxML tree must include bootstrap support values. At present the conclusions about STc95 and STc135 are not supported.

To improve phylogenetic support, we removed the draft genome from APEC SCI-07 and added 10 model InPEC strains. Our intent in this new selection is to show that AIEC STc135 and ExPEC strains phylogenetically close to BEN2908 have more similar orthologues than AIEC STc135 and InPEC strains. This is supported by the phylogenetic clustering of these strains, bootstrap values, Average Amino Acid Identity (AAI) comparisons, and consistent tree topology using different substitution matrices (available on github repository: [link](https://github.com/Martins-TW/BEN2908_Genome_Analysis.git))

6. Please specify the BLAST settings (identity, coverage thresholds, word size, or e-value) used to define the genomic regions (GRs), and justify the specific ≥4 kb cutoff, as this arbitrary threshold affects which loci are considered “GRs.”

BLAST was run with BLAST+ using default blastn parameters (word_size=11; reward=2; penalty=−3; gapopen=5; gapextend=2; e-value=10). BRIG visualization thresholds were set to 90% (upper) and 70% (lower) identity; these thresholds control intensity colouring of the ring (lines 221-223). As suggested in the comment below, BRIG alignment files containing coverage and other data were uploaded at my GitHub account ([link](https://github.com/Martins-TW/BEN2908_Genome_Analysis/tree/main/BRIG_alignments)).

The ≥4 kb cutoff was chosen for the following reasons:

First, it corresponds roughly to 3-4 genes given the typical gene density in *Escherichia coli* (~1 gene per ~1 kb; some references on NCBI: K-12 ([link](https://www.ncbi.nlm.nih.gov/datasets/genome/GCF_000005845.2/)), BEN2908 ([link](https://www.ncbi.nlm.nih.gov/datasets/genome/GCA_902705295.2/)), LF82 ([link](https://www.ncbi.nlm.nih.gov/datasets/genome/GCF_021398935.1/)). Thus, a 4 kb region is large enough to capture small functional clusters such as operons or adjacent co-functional genes, while still considering intergenic spaces and genetic elements that could not be identified by the characterization programs we used. Second, a 4 kb threshold has precedent in analogous genomic reports (e.g., genome announcements for APECO1 ([link](https://pmc.ncbi.nlm.nih.gov/articles/PMC1855855/)) and IMT5155 ([link](https://pmc.ncbi.nlm.nih.gov/articles/PMC4232414/))), which facilitates comparison with closely related *E. coli* strains (lines 224-228).

To complement, we also note that some previously described genomic islands in *E. coli* (for example, GimB (GR 25) and PAI-X (GR 4)) have <5 kb in size (S2 Table). So, adopting a cutoff larger than 4 kb could exclude these biologically relevant islands.

7. In addition, while Tables S1–S2 summarize GC content, coverage, and identities, the underlying files (BRIG project, FASTA sequences of each GR, and CDS functional annotation tables) should be deposited in a public repository (for example Github, to allow replication). If it is available, scripts should be public on GitHub or public repositories, or more detailed in a section of supplementary methods.

The BRIG alignment files, FASTA sequences of each GR, their annotations generated by RAST, the python scripts used, as well as EzAAI, RAxML, Orthofinder, Roary, PHASTEST outputs and the sequencing files mentioned in methods section, are available on GitHub at the following repository (lines 257-259): <https://github.com/Martins-TW/BEN2908_Genome_Analysis>

8. In the Table 2 and Figure they some plasmids are analyzed but they are not available for download. If pLF82 data were taken from an older publication, the comparative analysis is only partially reproducible; please provide the accession numbers or upload the sequence to a public repository.
The section was revised so that the analysis no longer relies on plasmid comparison with pLF82, which is not available for download. The information we included about pLF82 in Supplementary Table S7 was extracted from the description provided in the supplementary material of [Miquel et al. (2010)](https://pubmed.ncbi.nlm.nih.gov/20862302/).

9. For the functional assignments, please clarify the criteria for selecting “reviewed” vs “unreviewed” UniProtKB hits and provide the accession numbers of representative proteins. Without these details, other researchers cannot reproduce or verify the classification of features.

A better description of the reviewed vs unreviewed criteria was added to the “Ring Image generation and CDS functional characterization” Methods section (lines 243-247). The accession number of representative proteins from Uniprot database are available on Table 5 and S2 Table.

10. Please temper the VFAnalyzer/PHASTEST claims (from lines 221) by specifying identity/coverage thresholds, depositing the full outputs (Tables S4–S5 with raw hits), and avoiding absolute statements like “shares all” without qualification. I

PHASTEST full outputs were deposited in the GitHub repository and VFanalyzer full output is S5 Table with pathotype description and line coloring included. We also provided a description of how the programs were used in the “Genomic comparison and characterization of *E. coli* strains” Methods section (lines 179-186). The sentences were also modified to better attribute findings to the respective program outputs avoiding unqualified statements.

11. The same situation is in the plasmid section (from line 237) the description of pBEN2908 is super clear, but the statement that “all genes related to a ColV-like plasmid are present” is too absolute without showing the evidence in detail. A table listing each ColV-associated locus (e.g., iuc/iut, iro, etc) with accession, coverage, and identity values would be useful and also the Inc or MOB type groups in the same table.

As suggested, Table 2 now lists ColV-associated loci containing accession number, coverage, identity values, and PlasmidFinder identification. We also updated Figure 2 to include the same strains shown in Table 2.

12. In the Sugar Metabolism section (from line 328), please distinguish more clearly between functions previously validated in the literature (e.g., frz operon, GimA) and novel pathway predictions based on low-identity ORFs, so that readers can separate established evidence from putative annotations and similar in the identification of iron and metal uptake systems (from line 404), the phrasing “we identified systems related to metal uptake” should be revised to clarify that this refers to the detection of previously described operons through genome annotation, rather than novel experimental findings.

In the Adhesion and Invasion section (lines 412), please clarify that operons and loci (fim, auf, fdeC, ila) were detected through annotation rather than experimentally demonstrated, and temper the functional claims for ORF 1₁₁ as putative predictions based on moderate homology.

To clarify when the referred genes were already studied and when they were predicted, we divided each functional category (SM, Bf, A/I, …) into a Described and an Uncharacterized subsection. Also, we added a more careful statement when referring to low-identity ORFs, like ORF 1_6_, mentioned in the Sugar Metabolism section (lines 466-470). GR 11 (so, ORF 1_11_) was removed because we remade our alignments and found out that commensal K-12 have more than 50% of coverage and above 90% identity to GR 11. After this curation, 36 genomic regions remained absent from K-12 MG1655 and present in all strains from the ring (Fig. 3; S1 Table).

13. The Discussion is very detailed and in parts can be excessive. Several subsections (e.g., Sugar Metabolism, General Metabolism) repeat pathway-level explanations that could be streamlined or moved to Supplementary Material. A more concise discussion, with selective emphasis on novel findings, would greatly improve readability. Consider moving some of the descriptive detail into the Introduction (to set context) or into Supplementary Notes, while keeping the main text focused on the comparative insights.

As suggested, some of the text was removed from the discussion and some were modified to fit in the introduction.

14. In the Conclusion, please temper the statement on long-term conservation of uncharacterized ORFs, which is only inferred from two isolates 21 years apart, and explicitly clarify that all proposed functions remain hypothetical pending experimental validation.

As suggested, the statement was tempered and affirmation was broadened to clarify that these GRs are also present in several ExPEC strains, but require experimental validation to our predictive analysis (lines 769-772).

Minor revisions:

15. The statement that these findings “reinforce the importance in government monitoring of bacterial genomic evolution” (line 276) introduces a policy-oriented opinion that goes beyond the scope of the presented data. I recommend rephrasing this sentence to remain focused on the scientific evidence (i.e., the role of ColV-like plasmids in virulence).

Thanks for the comment, the suggested statement was removed.

16. Replace “softwares” with “software,” shorten long passive sentences.

done

17. Reframe causal or absolute statements (“shares all genes,” “ancestral proximity”) into cautious, data-supported wording (“shares X genes above threshold Y,” “phylogenetic proximity suggested but requires bootstrap support”).

Absolute statements were avoided, and wording was adjusted to reflect threshold-based interpretations.

Specific Comments

• Line 71: Please make explicit that you are now referring to human disease context.

added
• Line 93: Be careful with the term “cassette gene.” Are you referring to integron cassettes or simply modular groups of mobilizable genes? Clarify the terminology.

changed to genomic modules and gene modules

• Line 104: Explain or cite the “minor modification” of the ONT nanopore protocol. Without details, it is not reproducible.

Thank you for your comment. In fact, the minor changes are those indicated in the following sentences. The text has been amended as follows: Sheared DNA was End-Repaired using Oxford Nanopore recommendations for 1D Ligation sequencing (LSK-SQK 108), with minor modifications, as follows (line 129).
• Line 120: Please, provide the RAST version

done

• Lines 113–115: Wording issue: “The obtained raw data (.fast5 files) was base called…” → should be “The raw data were basecalled with Guppy v4.0.11 using [config].” Please also specify qscore filter and parameters.

The text was modified accordingly and the Guppy mode and qscore filter was specified (lines 139-142). The Nanoplot file generated comparing read quality before and after filtering was submitted to GitHub in the following repository: <https://github.com/Martins-TW/BEN2908_Genome_Analysis>

• Line 210–215: The statement that STc95 is closer to AIEC STc135 than to APEC SCI-07 cannot stand without bootstrap support. Please provide node support and, ideally, ANI/AAI values.

To improve phylogenetic support, we removed the draft genome from APEC SCI-07 and added 10 model InPEC strains. The new tree is supported by the phylogenetic clustering of these strains, bootstrap values, Average Amino Acid Identity (AAI) comparisons, and consistent tree topology using different substitution matrices (available on github repository; [link](https://github.com/Martins-TW/BEN2908_Genome_Analysis.git))

• Line 222–229: When describing shared virulence genes with LF82, avoid absolute terms like “all 76 genes.” Instead, specify thresholds for identity/coverage and include a full gene list in Supplementary Materials.

VFanalyzer full output is S5 Table with pathotype description and line coloring included. We also provided a description of how the programs were used in the “Genomic comparison and characterization of *E. coli* strains” Methods section (179-186). As suggested the sentences were also modified to avoid absolute terms.

• Line 300–311 (Table 3): Provide coverage/identity values for each “genomic region” absent or present, or the cut off used for declare as presence/absence.

S1 Table contains coverage and identity values to the 22 strains and cutoff was explicit in S1 Table comment and Table 3 title (lines 420-424). Also, in “Overview and metabolic functions of the GRs in common between ExPEC and AIEC strains” Results and Discussion section (lines 398-400).

• Line 461–466: The CRISPR comparison is interesting, but please provide a full spacer table with coordinates and system subtype classification to avoid confusion.

S3 Table was built to contain the requested information.

• Line 650–656 (Conclusion): The wording “attests the utility and importance” is too strong. Suggest softening to “suggests possible functional utility, subject to experimental validation.”
As suggested, we rephrased the conclusion to adopt a more tempered tone as follows: “In spite of that, this is a predictive work and experimental validation for these in silico analysis is necessary to define the true functionality of the novel ORFs identified” (lines 766-768).

Recommendation: Major revision. This is a very complex and beautifully crafted manuscript that will be a valuable reference once revised. With the additions of assembly QC, reproducibility deposits, phylogenetic support, careful terminology and parameters, the paper will fully meet the PLOS ONE publication criteria. Once these issues are addressed, the manuscript will meet the journal’s standards for transparency, reproducibility, and cautious interpretation.

Reviewer #2: I see the main contribution of this paper serving as a potentially valuable resource, providing a comprehensive genomic comparison of BEN2908 and related E. coli strains, and identifying candidate loci for virulence that warrant experimental validation. The current manuscript is mainly descriptive. Below, I list some comments that need addressing before I believe it is suitable for publication.
We sincerely appreciate the reviewer’s thoughtful and constructive feedback, which has greatly contributed to enhancing the quality of our manuscript.

Major Concerns

[1] Besides the strain of interest (BEN2908), 13 other E. coli strains are used to construct a phylogenetic tree. Instead of using the same 13 strains for the following analysis, the ring images are generated by comparing BEN2908 versus three other APEC strains, one AIEC strain (LF82), and one K-12 strain. Why only select a subset of the 13 strains for ring comparison?

Initially, we chose the two strains most closely related to BEN2908 (IMT5155 and APECO1) and the invasive AIEC LF82 to investigate overlapped regions with an invasive, but taxonomically distinct strain. K-12 was added as a commensal contrast. We also incorporated chi7122 because it clusters separately in the phylogenetic tree, giving us the opportunity to check for potential APEC specific regions, which showed no results.

However, to better align this comparison with the observed tree topology (Figure 1), we repeated the ring comparison using the strains phylogenetically closer to LF82/NRG857c and BEN2908, indicated by the grey arrows. These comprised two UPEC (CFT073 and 78-Pyelo), two NMEC (IHE3034 and RS218), two APEC (APECO1 and IMT5155), one AIEC (LF82) strain. The commensal strain K-12 was also added to identify regions absent in its genome. The strains UTI89 and NRG857c (also indicated with grey arrows) were not used in the ring comparison because they presented the same alignment pattern of RS218 and LF82, respectively (Figure 3).

[2] More details are needed to ensure the robustness of the phylogenetic analysis. When using MUSCLE, state whether ambiguous regions were trimmed and whether only the single-copy orthologs were included. For RAxML, report detailed bootstrap support values and/or compare the resulting topology under different substitution models.

Ambiguous regions were trimmed with trimAl and description was added to the “RAxML unrooted tree generation and average amino acid identity (AAI) to AIEC LF82” Methods section (lines 195-197). For the phylogeny, only single copy orthogroups were included (line 197-203). As suggested, we used different substitution models for generating the phylogenetic tree and used bootstrap to attest the topology in all of them. The 3,101 single copy orthogroups, and the phylogenetic trees made with different substitution models were submitted to the following GitHub repository in the Orthofinder and RAxML directories, respectively: <https://github.com/Martins-TW/BEN2908_Genome_Analysis>.

[3] When constructing the phylogenetic tree, is it possible to incorporate some enteric pathotypes, such as E2348/69 (EPEC reference strain) or EDL933 (EHEC reference strain)?

As suggested, we generated a new tree with the 10 genomes of different *E. coli* enteric pathotypes: four EHEC (O157:H7 Sakai, O157:H7 EDL933, 11368, 11128), three EAEC (42, 2009EL-2050, 55989), two ETEC (H10407, E24377A) and one EPEC (E2348/69). With this addition, we showed that AIEC strains, even though they are intestinal pathogens, are phylogenetically closer to extraintestinal pathogenic strains.

[4] Around 23 genomic regions are identified in common between STc95 model strains and LF82. Since NRG867c is often compared with LF82 as representatives of AIEC, how many of these 23 genomic regions are also detected in NRG867c?

NRG857c showed the same alignment pattern as LF82 (S1 Table), so we did not include it as it wouldn't add any new information to the image.

[5] When describing gene functions, I found it hard to distinguish functions predicted by KEGG from those demonstrated by experiments. The authors could emphasize functions that have been confirmed by both bioinformatics and bench.

To clarify when the referred genes were already studied and when they were predicted, we divided each functional category (SM, Bf, A/I, …) into a Described and an Uncharacterized subsection. We also removed parts of the discussion to focus on our findings.

Minor Comments

[6] Citation numbers 1 to 5 are missing in the main manuscript.

Thank you for pointing this out. The issue was due to a problem with the Word document and EndNote integration. We have carefully checked all the cited references and have made the necessary corrections to ensure that all citations are correctly included.

[7] Line 136, the software name “Orthofinder” should be “OrthoFinder”.

done

[8] Low figure resolution renders some key features unclear.

done

[9] The bibliography is extensive but not necessarily relevant. The authors should prioritize conclusions and related bibliography to emphasize the most novel findings.

We thank the reviewer for this comment. We cited 172 references to support different parts of the manuscript. In the Introduction, 39 were used to contribute to the contextual background; in Methods, 22 were used for the genome descriptions of each strain, 33 for the bioinformatic tools used (plus Liu et al. 2018 as the reference for our plasmid identification criteria), and 20 for previously described features (3 in BEN2908 and 17 reported in Table 4); in Discussion, 19 were used to support functional assignments of ORF homologs. The remaining 39 citations are distributed throughout the Discussion to support specific interpretations and comparisons.

Reviewer #3:

Thanks for your comments that were considered carefully and have contributed to the revision of the manuscript

Introduction
line 38/39 - a general definition of avian colibacillosis is needed to help appreciate the significance of the work. I understand that it is not well defined, but perhaps just a general definition/classification of the disease... AC is a major cause of mortality, systemic bacterial infection, affects X% of poultry, costs to poultry farmers, something like that. The authors give slightly more information in the second paragraph, but it would help the reader to move some of the information to the first paragraph.

As suggested, some information has been added. Prevalence of colibacillosis has not been studied worldwide and percentages varied a lot between countries. The associated costs have not been really calculated except in one study. Indeed, we added the following sentences:

Avian colibacillosis is characterized as systemic or localized infections that can affect birds of all ages and in all types of poultry production. It manifests in a range of clinical forms, including airsacculitis, pericarditis, perihepatitis, peritonitis, salpingitis and septicemia. It often results in high morbidity and mortality leading to economic losses in the industry throughout the world (e.g. in Netherlands, losses due to salpingitis were estimated at € 0.4 million, € 3.3 million and € 3.7 million for the layer-sector, the meat-sector and poultry farming, respectively) (lines 45-52).

line 65 - misplaced comma after "although". There are a few awkward sentences in the introduction and it should be re-read carefully and edited for clarity and ease of reading.

done

Line 87 - if the paper is about both BEN2908 and LF82, why is BEN2908 the only strain mentioned in the title and why is LF82 not mentioned more in the abstract?

Thanks for the comment. As suggested, we modified the Abstract and Title to refer to the new set of genomes analyzed.

General comment: I think the introduction could be re-structured for better flow of information, especially for the benefit of genomic science/bioinformatics readers who are not e. coli experts. There are some instances where information is initially referenced, but the context is not added until later (example- initial mention of AIEC in line 63 by way of comparing BEN2908, but then an explanation of the importance/clinical context of AIEC isn't mentioned again until the next paragraph on line 71. Other examples: ST complex information in lines 38 and 42-47 could be put into better context- a brief mention of the difference between STc and ST would be helpful for readers who are not intimately familiar with E. coli, but are interested in the paper for the comparative genomic aspect- as could the relationship between AIEC and ExPEC strains (paragraph starting on line 71).

We reconstructed the Introduction to better flow with the results obtained later. As suggested, we also added information regarding the difference of ST and STc in the Introduction (lines 66-68).

Methods
Genomic comparison: pathotype acronyms should be defined. APEC and AIEC are previously defined, but NMEC and UPEC are not.

done

Results
Line 202: what is the rationale for only looking at fimH and not other components of type 1 fimbriae, such as FimA?

FimH is the tip adhesin of type 1 fimbriae, being responsible for directly interacting with the host’s cell receptors. To clarify this, the following text was added to Assembly, characterization and phylogenetic analysis of BEN2908 section of Results and Discussion : “*fimH* is commonly used as T1F typing gene because it directly mediates attachment to host cells, and it has been shown that its allelic variation alters affinity for mannosylated receptors, affecting adhesion and invasion.” (lines 287-290)

Line 208: the mention of x7122's sequence similarity to K12 feels distracting from the manuscript's goal of comparing BEN2908 to the rest and from the stated goal of the paragraph (in line 198).
We agree and removed the sentence, the text now emphasizes the phylogenetic proximity of BEN2908 and related strains to AIEC LF82 and NRG857c.

Line 212: missing word "strains" after STc95 and before "(fumC38, mdh17, and recA26)".
The section was modified and this statement was removed from the paper.

Line 224-226: awkward sentence structure (and grammatical error) starting with "if considered (considering) exclusively".

The section was modified and this statement was removed from the paper.

Line 234: "results from the PHASTEST program went in the other direction" should be rephrased to clearly state the result shows a lack of commonality rather than using casual language and asking the reader to infer the result.

As suggested, this was rephrased as follows: “In contrast, PHASTEST results diverged…” (lines 333-335)

Line 238: please state the plasmid sizes in the text in addition to their location in the table.
Plasmid sizes were added to Table 2 and S7 Table.

Line 238: it might be interesting to also state what genes are found on the larger plasmids that are not found on pBEN2908.

We revised this section and removed the comparison based on plasmid size to focus on ColV-like content (see Table 2). Still, plasmid characterization remains available in S7 Table.

Line 239: define and describe the characteristics of a ColV-like plasmid. Colicins are not mentioned in the text prior to this point, nor are they addressed/explained in this paragraph in a meaningful way.
The section “pBEN2908 is a ColV-like plasmid” was modified to describe and define ColV plasmids and their importance in the beginning of the section: “Colicin V (ColV) plasmids have been shown to play a pivotal role in the pathogenesis of extraintestinal pathogenic *E. coli*. Its name was attributed to the production of colicin V, a bacteriocin originally described by Gratia in 1925 as “factor V” (100). These plasmids usually range from 80 to 180 kbp and were identified in a variety of E. coli hosts, with particular significance in poultry infections (100-104).” (lines 337-341).

Line 240: the authors state that "all genes related to a ColV-like plasmid are present on pBEN2908", but these genes are not explicitly highlighted in the text or in Figure 2. Please add an indication of which genes these are, either in the text or in the figure.

The section was modified to explicitly describe these genes (lines 344-352), and both Table 2 and Figure 2 were updated to include them.

Line 241: "APEC plasmids are also known to encode various virulence factors" please confirm in the text that these are not only "known to be encoded" but ARE, in fact, encoded in the plasmid of interest.
The section was updated to state these confirmations more explicitly.

Line 265: "pO83-CORR plasmid of strain NRG857c also carries all of the ColV‐associated genes" is this expected based on its ST, etc? Please note the ST or pathotype or such in the text so that the reader does not have to frequently flip back to Table 1 to look at the most important details and understand why this specific strain is being discussed for comparison.

As suggested, ST and pathotype information were added to the new Table 2 and pathotype is now mentioned in the text (line 361). We also added in the ending of the section that the presence of a ColV-like plasmid in AIEC NRG857c is noteworthy, as it suggests a potential APEC-derived zoonotic trait (lines 365-367).

Line 273: this level of significance would be great earlier on in this section of the results to help explain the significance of ColV plasmids. Include something like this up front to address my earlier comment on line 239.

As suggested, the plasmid significance was moved earlier on the section (lines 337-344).

Line 328: remove "the" at the beginning of the sentence. Start with "Genomic regions....". This also happens throughout the next few paragraphs- the authors switch back and forth between referring to GRs as "the GR #" or "GR #". Please choose one convention and stick with it, though I recommend removing "The" for ease of reading.

As suggested, GR was used without “the” throughout the text.

Line 336: The reference to "the authors showed" on this line is not clearly connected to citation #21.
The sentence was rephrased, and the citation was placed closer to it (line 444).

Line 338-341: awkward sentence phrasing.

Sentence rephrased

Line 352: please change "Aside from those" to more conventionally professional language, such as "Additionally".
As suggested, “aside from those” was changed to “additionally”

Line 355: What is the significance of the catabolism of threonic acid to this paper that warrants its specific inclusion? Please elaborate. This paragraph in general is missing an explanation of the named genes' functions, unlike the surrounding paragraphs which do include that information.
The sentence referring to threonic acid catabolism was removed to better focus on the role of PLP cofactor synthesis (lines 456-459), which is directly connected to the metabolic functions discussed later in the general metabolism section.

Line 380: "pathway" should be plural.

done

Line 382: Potential rephrase, instead of using vice-versa: "...named UxuB, catalyses the reversible interconversion of..."

Modified as suggested.

Line 426: The discussion of the interaction between Fim proteins does not include the function of FimH. Why, then, is this the Fim protein being analyzed (see also the comment on line 202)?
As noted above, *fimH* characterization is a common typing scheme in *E. coli* because of its direct interaction with host receptors. To keep the focus of this section clear, the segment about *fimH* was removed.

Conclusion
The limitations of the study should be acknowledged when drawing conclusions.
The limitations of the study were acknowledged in the conclusion, reinforcing that this is a predictive work and requires experimental validation (lines 766-768).

General comment: Casual and/or clunky language is used throughout the text. It should be carefully reviewed to ensure professionalism in the writing. Overall, the science is sound and the analysis is satisfactory.
Instances of "[author] and coll." should be edited to "[author] et al"

The language throughout the manuscript was revised for clarity, and all instances of “[author] and coll.” were corrected to “[author] et al.” as suggested.

Reviewer #4: The manuscript “Sequencing of the Invasive E. coli Strain BEN2908 Isolated from Poultry: A Comparative Investigation of Genomic Regions Shared with Other Invasive Model
Strains” by Martins et al., provide insight into sequence similarity between a invasive strain BEN2908 from poultry and compares it with previously reported E. coli genomes. The study identified sequence diversity as well as conserved regions in the genome and its biological significance. Following are the minor comments on the manuscript.

We appreciate the reviewer’s feedback, which has been useful in refining the manuscript.

1. References for the genes that contribute to the virulence of E. coli (line 280) should be provided
The following references were added: [Schouler et al. (2012)](https://journals.asm.org/doi/full/10.1128/jcm.05057-11), [Mitchell (2015)](https://pubmed.ncbi.nlm.nih.gov/25480753/), and [Denamur et al. (2020)](https://pubmed.ncbi.nlm.nih.gov/32826992/) (lines 384-385).

2. In line 335, reference 21 appears in italics, and reference 153 is also italicized; please ensure consistent formatting.

Done

3. The references numbered 154–159 are listed in the reference section but not cited in the main text.

Thank you for pointing this out. We have carefully checked all the cited references and have made the necessary corrections to ensure that all citations are correctly included.

4. At line 609, the temperature unit is incorrectly written as ‘Celsius’; it should be expressed in °C, consistent with the formatting used throughout the manuscript

Done

5. Please provide a reference for the previously described GRs in E. coli mentioned in line 413.

Citations required are listed in Table 4. We thus quoted Table 4 after mentioning the already described GRs (line 522).

6. Please include an appropriate reference to support the statement in line 531.

We cited Nakayama et al, 1994 (<https://www.jbc.org/article/S0021-9258(17)43626-X/pdf>), cited also in Table 4.

Reviewer #5: The authors have sequenced and compared BEN2908 genome with other model genomes of ExPEC strains and come up with some features that were unique to BEN2908, that is not shared with other genomes, that may contribute to pathogenesis.

We acknowledge the reviewer’s constructive comments, which have helped improve the manuscript

Introduction: In the introduction, the authors describe the limited understanding of the pathophysiology of avian colibacillosis. In this context, they have chosen ST95 strains, particularly BEN2908, a well-studied strain. However, it is unclear why they also selected strain LF82—an adherent-invasive E. coli (AIEC) commonly associated with Crohn’s disease in humans—for comparison. Given that the focus of the study is the characterization of an extraintestinal pathogenic E. coli (ExPEC), what was the rationale for including a strain that is primarily an intestinal pathogen?
The rationale was to show that the highly invasive BEN2908 is genetically close to AIEC strains from the ST135, which suggests that Intestinal invasive strains share some of the same virulence mechanisms seen on ExPEC. We modified the introduction to describe clearly common pathogenic traits among these strains, such as T6SS, metal uptake systems, and virulence factors, such as Vat protease (lines 97-108). This highlights the zoonotic potential of APEC, which is further suggested by the identification of the ColV plasmid in AIEC NRG857c (lines 365-367).

Methods 1: If the genome sequencing was outsourced to Genome and Transcriptome Facility at Bordeaux, France - technical details can be reduced to minimal.

The technical details described in this section are the minor modifications from the protocol. To better state this, we changed the sentence to “Sheared DNA was End-Repaired using Oxford Nanopore recommendations for 1D Ligation sequencing (LSK-SQK 108), with minor modifications, as follows.”(line 129)

Methods 2:The genome of BEN2908 strain was submitted in 2022 (The two accession numbers: LR740776.1; LR740777.2). This study has compared BEN2908 genome with 13 other genomes. The comparison was done with four APEC, two AIEC, two NMEC, three UPEC and two commensal E. coli strains. What was the rational to compare against just these categories?

The rationale was to show that the AIEC strains are phylogenetic close to STc95 ExPEC strains. To evidence this proximity, we added 10 studied genomes of different InPEC (Intestinal Pathogenic *E. coli*) pathotypes: four EHEC (O157:H7 Sakai, O157:H7 EDL933, 11368, 11128), three EAEC (42, 2009EL-2050, 55989), two ETEC (H10407, E24377A) and one EPEC (E2348/69). With this addition, we showed that AIEC strains, even though they are intestinal pathogens, are phylogenetically closer to the extraintestinal pathogenic strains near BEN2908 (Figure 1, grey arrows).

Results & Discussion

1. The authors could have incorporated a larger set of genomes readily available from public databases, rather than limiting the analysis to just 14 genomes representing various ST types, pathotypes, and serotypes. This limited selection may reduce the resolution of the phylogenomic tree and the accuracy of virulome comparisons. The authors could have included a larger set of genomes and compared with a pangenome approach to get a wider and rather better picture on the virulome of strain BEN2908.

As suggested, we broaden our phylogeny to contain the genomes of 10 studied intestinal pathogenic strains from four pathotypes, resulting in nine distinct pathotypes in total (ExPEC: APEC, UPEC, and NMEC; InPEC: AIEC, EHEC, EAEC, ETEC, and EPEC; and Commensal).

Regarding the pangenome analysis, our goal was to identify genomic regions common to BEN2908, AIEC LF82/NRG857c, and other extraintestinal pathogenic strains close to them in the phylogenetic tree (grey arrows) and obtain information about their content.

Considering that some of this content consists of novel genes, a pangenome analysis alone would not provide a fine description of them, since it is limited by the annotation programs used, which often do not give sufficient information about uncharacterized genes. Therefore, we thought it would be better to investigate these regions using the ring comparison and characterizing each CDS individually through domain analysis (CD-Search) and sequence homology (BLASTp).

Still, we considered it informative to run Roary to compare core genome content: Roary showed that the core genome shared between AIEC and ExPEC strains is larger than that shared between AIEC and InPEC strains, further indicating a closer relationship between this extraintestinal group and AIEC (lines 323-325). From this result we generated a Venn diagram, which we made available in the following GitHub repository together with the raw analysis outputs: <https://github.com/Martins-TW/BEN2908_Genome_Analysis>

2. Fig 2 – Compares only plasmids of p1ColV5155 (IMT5155), pAPEC-O1-ColBM (APEC O1), and pAPEC-1( 7122) with that of BEN2908. What about plasmids from other genomes?

The plasmid section was revised to focus on the comparison of all ColV-like plasmids identified across the 23 strains. For this reason, the ColV-like plasmid from NRG857c (pO83-CORR) was also added to Figure 2.

3. Page 21: lines 284-286 - The BEN2908 genome was set as the reference and five other strains as subjects: three model APEC strains (IMT5155, APEC O1, 7122), the commensal K-12 strain MG1655, and AIEC strain LF82. Why were only these genomes compared?

Initially, we chose the two strains most closely related to BEN2908 (IMT5155 and APECO1) and the invasive AIEC LF82 to investigate overlapped regions with an invasive, but taxonomically distinct strain. K-12 was added as a commensal contrast. We also incorporated chi7122 because it clusters separately in the phylogenetic tree, giving us the opportunity to check for potential APEC specific regions, which showed no results.

However, to better align this comparison with the observed tree topology (Figure 1), we repeated the ring comparison using the strains phylogenetically closer to LF82/NRG857c and BEN2908, indicated by the grey arrows. These comprised two UPEC (CFT073 and 78-Pyelo), two NMEC (IHE3034 and RS218), two APEC (APECO1 and IMT5155), one AIEC (LF82) strain. The commensal strain K-12 was also added to identify regions absent in its genome. The strains UTI89 and NRG857c (also indicated with grey arrows) were not used in the ring comparison because they presented the same alignment pattern of RS218 and LF82, respectively (Figure 3).

4. With the BRIG analysis, BEN2908 was used as the reference genome and other genomes were compared to the reference genome. Comparing BEN2908 with other reference genomes would have given better insights of what BEN2908 was lacking.

Our aim was to identify genomic regions of BEN2908 in other strains, because of its invasiveness. To broaden the BRIG comparison, we expanded the ring analysis to include all genomes phylogenetically close to LF82 and BEN2908 (marked with grey arrows on the tree), so the figure now better highlights regions common to these related strains.
