# Supplementary material for: Sequencing of the invasive E. coli strain BEN2908 isolated from poultry: A comparative investigation of genomic regions shared with intestinal and extraintestinal model E. coli strains
Source: PLoS One. 2026 Feb 23;21(2):e0342894. doi: 10.1371/journal.pone.0342894 (PMC12928451; doi:10.1371/journal.pone.0342894)
Supplement: Attachment — Submitted filename: response reviewers PONE-D-25-49371R2 final.docx [file pone.0342894.s012.docx]

**Response to reviewers:**

**Reviewer #1:**

I reviewed the revised submission together with the authors’ responses. Overall, the revision is clearly improved compared with the previous version. The manuscript reads more coherently, the methods are more reproducible (tool versions and key phylogenetic settings are now provided), and the comparative genomics results are presented in a clearer narrative. The authors have addressed most of the substantive concerns raised previously.
That said, there are still several points that should be corrected before acceptance.

We thank you for your thorough review of our document, which has enabled us to correct the remaining errors

**Major points**

1) Inconsistency in T6SS genomic region (GR) assignment.
In the “Secretion systems” section, the authors report T6SS in GR 11 and GR 19, but later state: “One set containing more than fifteen CDS … were found occurring in GR 20.” This does not match Table 4, where the larger T6SS cluster corresponds to GR 19 and modules are present in GR 11. Please verify and correct the GR numbering in the text (very likely GR 20 should be GR 19) so that the manuscript is internally consistent.

Indeed, in this case GR 20 was GR 19, thanks for the comment. The text was modified accordingly. (line 613).

2) The Illumina read retention after filtering is extremely low and needs clarification.
The manuscript reports 32,274,430 MiSeq reads but only 1,713,025 reads retained after trimming and quality filtering. This is an unusually large reduction (approximately 95% discarded). This may reflect stringent filtering or an underlying quality issue, but the manuscript should explicitly state the filtering criteria (quality threshold, minimum length) and clarify whether counts refer to reads or read pairs. A short justification would help readers interpret this and reduce concern about data quality. Please clarify in methods the execution environment and provide version/parameters accordingly of “fastq_quality_filter”.

Thank you for your observation. Indeed the number was mistaken; it was actually 3,227,430 reads and not 32,227,430. To clarify each step of the processing, the text was modified to attend your considerations: “Illumina reads were trimmed for adapters and low-quality bases using Trimmomatic (v. 0.32)(38), with the following parameters: ILLUMINACLIP:TruSeq3-PE.fa:2:30:10; LEADING:30; TRAILING:30; HEADCROP:20; MINLEN:150. This resulted in 2,476,272 paired reads with minimum length of 150 bp, which were further filtered with a Q20 cutoff using fastq_quality_filter (v. 1.0.0), available on Galaxy platform (v. 25.0)(39). After filtering, a total of 1,713,025 reads were retained, yielding a coverage depth of 282x and breadth of 99.86%, as assessed by BWA (v. 0.7.19)(40).” (lines 148-153)

3) Define explicitly what “absent in MG1655” means.
The ring comparison is built around calling genomic regions “absent in MG1655,” but the manuscript does not provide a clear operational definition of absence ( for example, coverage and identity thresholds). Since the authors already define inclusion thresholds in Table 3 (>50% coverage; “partial” <70%), the same explicit definition should be given for “absence. (pages 27 and 36)
An explicit definition of absence was added to lines 239 and 411.

**Minor points**

Page 24: “Read quality and length distributions were then assessed using.” Has an extra “.”

done

Page 36: (line 389) Double colon (“subjects: : two APEC”)

done

Page 29: Double period in Data availability (“LR740777.2. . Raw reads”)

done

Harmonize strain and plasmid naming conventions (APEC O1 vs APECO1). The manuscript alternates between “APEC O1” and “APECO1” (including in table headers). Please adopt a single convention for strain names and plasmid names and apply it consistently throughout.

done, APEC O1 was defined as the convention.

Page 30: Table 1, rephrase the fimH2343 footnote for clarity. The note stating that fimH2343 results in the fimH5 translated amino acid sequence is difficult to interpret as written and mixes allele nomenclature with protein identity. Please rewrite this footnote more precisely (nucleotide change and resulting amino acid equivalence).

We agreed with your comment and the footnote was edited as follows:”¹ The allele fimH2343 differs from fimH15 by a single nucleotide mutation (537 G>A), resulting in a non-synonymous substitution on the amino acid sequence (180 G>S). (lines 293 and 294)

Page 44: Maintain consistent framing of homology-based inferences. In a few places, the wording still suggests experimental identification rather than annotation and or prediction. Minor edits (“annotated,” “predicted,” “detected operons involved in…”) would better align with the evidence (especially for metal uptake and pathway reconstruction).

To make a clear distinction between predicted and experimental identification we modified some paragraphs and made wording edits (lines 458-468, 476-479, 484-485, 531, 544, 566, 581, 601, 630, 632, and 786).

Standardize the way the ring comparison strain set is counted. At one point the manuscript refers to “7 strains phylogenetically close,” while elsewhere it describes a comparison set including eight strains (plus a commensal reference). Please standardize the counting and wording to avoid confusion.

To avoid confusion regarding the phylogenetically close strains we made wording edits (lines 311, 323, and 407).

Add a brief limitation statement for the metabolic pathway reconstruction. The KEGG-based reconstruction is plausible, but it would be useful to add one sentence noting that this is inferred from homology-based KO assignments and requires experimental validation of regulation and transport specificity.

We agree with your comment, and an explanation was added to the ”Genomic comparison and characterization of *E. coli* strains” Methods section: “The KEGG Pathway (KP) and KEGG Orthology (KO) databases were used by mapping the KO assignment numbers of the uncharacterized ORF homologs identified in this work onto KP maps. This allowed us to predict metabolic pathways potentially related to the molecular functions of the novel ORFs. However, these predictions are based on homology-derived KO assignments and require experimental validation of protein activity, regulation and specificity” (lines 187-192)

**Recommendation**

This is a strong revision and I think it can be accepted after the authors address the points above. The remaining issues are mainly about internal consistency (notably the T6SS GR numbering), interpretability/reproducibility (explicit thresholds; Illumina filtering and read counts), and copyediting (punctuation and terminology). Addressing these items will strengthen the manuscript and reduce the risk of additional questions in a subsequent round.

**Reviewer #2:**

Thank you for addressing all my concerns and comments. Congratulations on the manuscript. I would appreciate it if the figures could be provided at a higher resolution.

We thank the reviewer for the comment. Regarding the figures resolution, the PDF with the figures sent to you is generated by PLOS ONE during the submission process, and we have no control of the quality over this step. Nonetheless, as suggested, we improved the quality of the figures.
